# Current developments in artificial intelligence from obstetrics and gynecology to urogynecology

**DOI:** 10.3389/fmed.2023.1098205

**Published:** 2023-02-23

**Authors:** Mehmet Murat Seval, Bulut Varlı

**Affiliations:** Department of Obstetrics and Gynecology, Ankara University School of Medicine, Ankara, Türkiye

**Keywords:** artificial intelligence, deep learning, machine learning, urogynecology, urinary incontinence, pelvic organ prolapse

## Abstract

In today’s medical practice clinicians need to struggle with a huge amount of data to improve the outcomes of the patients. Sometimes one clinician needs to deal with thousands of ultrasound images or hundred papers of laboratory results. To overcome this shortage, computers get in help of human beings and they are educated under the term “artificial intelligence.” We were using artificial intelligence in our daily lives (i.e., Google, Netflix, etc.), but applications in medicine are relatively new. In obstetrics and gynecology, artificial intelligence models mostly use ultrasound images for diagnostic purposes but nowadays researchers started to use other medical recordings like non-stress tests or urodynamics study results to develop artificial intelligence applications. Urogynecology is a developing subspecialty of obstetrics and gynecology, and articles about artificial intelligence in urogynecology are limited but in this review, we aimed to increase clinicians’ knowledge about this new approach.

## Introduction

**Figure 1:**
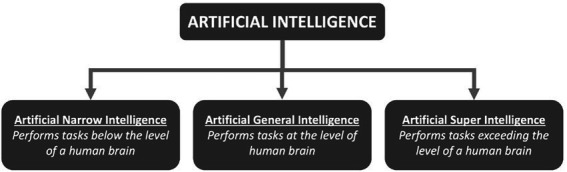
The three types of artificial intelligence.

In contrast to natural intelligence produced by animals, including humans, artificial intelligence (AI) refers to the intelligence demonstrated by computer systems. We use AI in daily life routinely, with web search engines (like Google), recommendation systems on online entertainment platforms (YouTube and Netflix), speech recognition (Siri and Alexa), and self-driving cars (Tesla) are some examples. In 1956, John McCarthy organized the first academic meeting on the subject at Dartmouth College, coining the phrase “artificial intelligence.” Artificial intelligence can be categorized in three headings (Figure 1).

### Artificial narrow intelligence

It is possible to develop problem-solving skills using text, audio, or image data. A processor could easily complete a single task at this level of AI. In spite of AI’s supremacy in chess, it has an IQ of just 0 (zero).

### Artificial general intelligence

At this degree of artificial intelligence, an intelligent creature can fully comprehend all complicated tasks in the same way that an individual can.

### Artificial superintelligence

While humans will never attain this degree of intelligence, machines may be able to surpass humans in terms of intelligence and behavioral skills.

## Working mechanism of artificial intelligence

### Machine learning

Human brain-like capabilities for AI are mostly achieved *via* machine learning. Machine learning uses a big amount of data to figure out how factors interact with each other. Supervised, unsupervised, and reinforcement learning are the three main approaches of machine learning (Table 1). To begin the supervised learning method, the input data must first be labeled. This is a great method for classifying and calculating regressions. Algorithms are designed to use this method because we know exactly what they should learn, and it’s the most popular method of training by far.

**Table 1:** The three main machine learning techniques.

Supervised learning	Unsupervised learning	Reinforcement learning
- Learning with a labeled training dataset	- Discovering patterns in unlabeled data	- Learning based on feedback or reward
- Predict surgical site infection rates after cesarean section: Build a regression model by providing data on the patients’ characteristics and surgical site infections to show variables that best correlate with infections	- Categorize ultrasound examination by normal or abnormal images: Use deep learning techniques to build a model that learns different features of images to recognize different patterns	- Allocate specialized medical services for various obstetric emergency patients

For unsupervised learning, there is no requirement for labeled data, in contrast to supervised learning. In data analysis and the generation of novel hypotheses, it is routinely used to uncover patterns from the data that could otherwise go undetected (1).

It is possible to think of reinforcement learning as a hybrid between unsupervised and supervised. It allows the model to learn how to complete the tasks *via* a sequence of decisions without being explicitly instructed on how to do so.

### Deep learning

Deep learning is a subset of machine learning and artificial neural networks mimic the human brain’s ability to make automatic predictions from training data sets using numerous layers of artificial neuronal networks. Deep learning refers to the use of numerous layers of filters, each of which provides an output score that serves as the input for the following layer. Table 2 describes the key differences between machine learning and deep learning.

**Table 2:** Differences between machine learning and deep learning.

Machine learning	Deep learning
A subfield of artificial intelligence	A subfield of machine learning
Considerable human intervention is needed to correct and learn	Learns by itself from experiences and prior failures
Able to learn with less data sets	Need a significant quantity of data
Shorter training time and lesser accuracy	Longer training and higher accuracy
Establishes basic, linear relationships	Generates complex, non-linear relationships

## Artificial intelligence in obstetrics and gynecology

Artificial intelligence with its capabilities will enable a much more effective and efficient healthcare system. AI can aid in the determination of diagnosis and prognosis, therapy optimization, and drug discovery. Artificial intelligence is becoming popular in the field of obstetrics and gynecology rapidly. At the 29th World Congress of the International Society of Ultrasound in Obstetrics and Gynecology (ISUOG) in 2019, 14 abstracts explicitly addressing AI were presented, on the other hand, a total of 13 abstracts were presented at the previous six ISUOG World Congresses (2013–2018).

Ultrasound images for nearly all situations in obstetrics and gynecology are available. As a result of this large database, AI applications for perinatal medicine and gynecologic oncology were reported (2–12).

The great majority of the research relied on ultrasound images, but physicians also tried out AI-based cardiotocography analysis for intrapartum fetal monitoring (13). Idowu et al. (14) emphasized the importance of using artificial intelligence to detect preterm labor. Electrohysterography signals were used in this study, and they were identified using three different machine-learning algorithms to help detect true labor and reliably diagnose premature labor (14).

Couples are being referred for assisted reproductive therapies at an increasing rate, and AI solutions in this field are gaining ground daily. Guy and colleagues used data mining and artificial intelligence to create a computer model that can help clinicians in the prediction of pregnancy after *in vitro* fertilization (IVF). Data mining (DM) is a technique for uncovering patterns in large databases that combines artificial intelligence and advanced statistics. DM obtains the needed data and is also capable of finding other important factors that may influence the outcome, hence increasing the amount of data that can be used (15). Manna et al. (16) suggested combining AI and ANNs to extract texture descriptors from oocyte or embryo images, with this method AI can identify the most viable oocytes and embryos with a high chance of pregnancy.

Neural network models are being used to estimate prognoses for patients with ovarian cancer. In a report, Enshaei et al. revealed that ANNs could predict survival with a 97% accuracy (17). It can also predict which treatment will be most beneficial for each patient depending on their diagnosis. Researchers at Brigham and Women’s Hospital and Dana-Farber Cancer Institute used AI to manipulate vast volumes of microribonucleic acid (RNA) data to build models capable of diagnosing early ovarian cancer (17). In comparison to an ultrasound screening test, which detected abnormal results less than 5% of the time, the AI neural network could keep up with the intricate linkages between microRNA and accurately recognized nearly 100% of anomalies associated with ovarian cancer (18). Additionally, AI outperformed human experts when it came to analyze pre-cancer images of the cervical region. Deep learning algorithms based on artificial intelligence can collect a large number of photographs related to cervical cancer screening and reliably detect diseased tissue. Patients can be treated on the same visit because minimum training is required and the results are obtained quickly (19).

Physical AI has been employed in surgery more frequently than virtual AI. Virtual AI forecasts the outcome using established patient parameters, repeating patterns, and treatment algorithms, as opposed to the surgical field, which has a high number of independent variables. The consistency of different tissues, the surgeon’s skill level, the changes made to the surgical field while operating, and a unique variation between patients and their pathology are only a few of these variables; ultimately, these distinct characteristics make designing an algorithm challenging (20). In various ways, AI has helped in gynecological surgery, including imaging and spatial awareness. Artificial intelligence can aid surgeons by enhancing imaging both before and during surgery. Three-dimensional (3DP) printing that mimics the operation site is far superior to two-dimensional (2D) printing because it provides a more accurate picture of the actual mode (21). This enables more exact preoperative planning, realistic trainee practice, and previously impossible preoperative planning.

Additionally, AI has contributed to the reduction of operating time and precision, resulting in fewer operative issues. Such augmented reality is used to accomplish this. A computer reconstructs real-world items and digitally enhances them to generate a more informative visual representation in augmented reality. While the system has several drawbacks, it also has some advantages, such as increased precision, safety, and a reduction in the time it takes to execute tasks (22). AI has aided in the development of spatial awareness, notifying surgeons when vital vessels or structures are hidden, allowing them to be recognized quickly and vital structures to be protected. One example is isolating the ureters during gynecological surgery. An endoscopic system powered by artificial intelligence was used in a study to detect the depth and position of the ureters using algorithms, displaying greater accuracy and safety (23).

## Artificial intelligence in urogynecology

As is the issue with many other fields of medicine, the use of AI in urogynecology is still experimental, and the optimal regions for AI in urogynecology are not clearly specified; but we will endeavor to summarize the current understanding regarding the use of AI in urogynecology.

To begin, advances in artificial intelligence allow telemedicine to advance. Patients’ conditions might be remotely monitored, tracked, and regulated with the help of wearable devices linked to AI systems. Electronic medical records software currently automates caregiver scheduling, organizes care plans, provides follow-up alerts, and automates payment, as well as offering patient and family portals. Virtual visits allow patients who live far away or have restricted mobility to receive follow-up care without having to undergo a physical examination, which can cut wait times. Virtual visits are becoming more common, particularly during the peak days of the COVID-19 pandemic. Wearable devices like urine incontinence monitors and post-void residual bladder volume scanners could help telemedicine in urogynecology. Wearable bladder volume monitors have recently been developed for use in children with nocturnal enuresis and other types of urinary incontinence (24). To monitor patients, treating teams may be able to access collected data remotely, or this may someday be done by an AI system.

Deep learning may be used to recognize medical pictures, as previously indicated, and Oral et al. (25) evaluated the role of dynamic MRI in the diagnosis and quantification of POP. From a midsagittal aspect, they looked at the 15 dynamic MR images. Although the writers did not appropriately identify the stage of prolapse in the publication, these pictures portray patients at varying levels of prolapse who had not previously received POP surgery. For all MRI reference sites, the semiautomated pelvic floor measurement method produced exceptionally consistent and exact placements. Furthermore, the model detects reference locations quicker than the old method. In the following years, Nekooeimehr et al. (26) proposed a method for automatically monitoring and segmenting pelvic organs on dynamic magnetic resonance imaging (MRI), followed by multiple-object trajectory classification, to aid in the understanding of pelvic organ prolapse (POP). According to their findings, the current method is capable of autonomously tracking and segmenting pelvic organs in 94 cases with a Dice similarity score of more than 78% and a Hausdorff distance of 5.2 mm. Statistically significant associations between various radiomic markers and urodynamic findings were discovered by Keene et al. The authors concluded that CT texture analysis of the bladder wall could be a useful tool for identifying patients with high-risk urodynamic features in spina bifida and that it could be employed instead of or in addition to urodynamics in neurogenic patient populations in the future (27). To reduce human interference and standardize urodynamic tract interpretation, Wang et al. (28) identified detrusor overactivty (DO) patterns linked with clinical findings. They used manifold learning (29, 30) and dynamic time-warping techniques. They reported an overall accuracy of 81.35%, a sensitivity of 76.92%, and a specificity of 81.41% for their AI-based model for identifying the DO. Cullingsworth et al. (31) focused examined the frequency and amplitudes of low-amplitude rhythmic detrusor contractions (LARC) in patients with DO and discovered a subset of them. The model was automatically constructed using a Fast Fourier Transform approach (32) that yielded 100% specificity. This level of precision was obtained while LARC was recognized independently of abdominal pressure traces, paving the way for future automated urodynamic trace interpretation. Niederhauser et al. (33) used ‘Wavelet’ time-frequency analysis in conjunction with AI-based algorithms to determine the existence of various subgroups of overactive bladder (OAB) patients based on the amplitude and frequency of non-voiding contractions and to investigate the possibility of automatically detecting DO in the third promising study on machine learning-based urodynamic automation. They were successful in identifying various OAB subgroups, and their machine learning-based algorithms for DO detection functioned well. There is a scarcity of information about AI’s therapeutic implications of in urogynecology. Whangbo and colleagues developed a device that can be used instead of a voiding diary. This is a smart bracelet that uses the patient’s particular postures to identify the time and the intervals between micturition. The voiding diary is the gold standard for examining patients with lower urinary tract symptoms, but it takes time and necessitates that patients stay home. This technology will provide data on actual VD and ease clinical evaluation (34).

In a cohort of 559 female patients treated with anticholinergic drugs for OAB, Sheyn et al. (35) built and externally validated a prediction model for anticholinergic response using an RF algorithm. The model performed brilliantly (sensitivity 80.4%; sensitivity 77.4%). This study relied on a subjective report of successful treatment, regardless of its good outcome.

Although it is generally intended by the surgeons on the field of urogynecology, the reconstruction of pelvic anatomy with surgery may not always improve health related quality-of-life (QoL) in all women with pelvic floor dysfunctions. Thus, predicting reduced health related QoL after a surgical procedure for a pelvic floor disorder provides the opportunity for individual targeted therapies of dysfunctions and disorders. Including symptom and quality-of-life scales in AI systems that evaluate the diagnosis and follow-up progress after treatments seem to be promising for the accurate and effective management of pelvic floor dysfunctions. However, these studies were limited in the literature and this issue should be investigated in future studies.

## Challenges of the artificial intelligence

While there are many potential benefits to introducing AI into clinical practice, there are also many challenges and unknowns that may cause worry. The quantity and quality of the data used to generate the models is the fundamental constraint on the robustness of these methods. This is particularly true for rare disorders that have erratic or subjective symptoms. As a result, we have been asked to automate data collection and quality control to get more powerful tools. Medical software, electronic medical records, care platforms, ambulatory devices, patient parameters, surveys, and measures of patient-related outcomes should all be developed in collaboration. Finally, ethical means for archiving surgical and perioperative data must be devised. The collection of high-quality data that can ultimately help patients requires standardization of methods across broad territorial groups and coordination among institutions.

When applying AI in clinical practice, another factor to consider is its applicability. Non-mentioned clinical data are ignored by the AI model because AI models can only account for data that were ‘seen’ during training. As a result, a growing segment of healthcare for AI research is committed to constructing AI models that integrate imaging and electronic health record data to enable accurate diagnosis and treatment.

When using machine learning in a clinical setting, the most important issue to overcome is trust, which arises when clinicians and patients accept the system’s recommendations. The data is noisy, sophisticated, multidimensional, and skewed toward the catchment area of the founding hospital systems, where the model was created. Another issue is the possibility of professional liability for doctors who use AI. Should hospitals and doctors be held liable for judgments made by artificial intelligence software?

## Conclusion

Throughout the last decade, excitement for artificial intelligence has increased massively. Numerous recent studies have focused on the potential of artificial intelligence in urogynecology not only to investigate the pathophysiology of lower urinary tract dysfunction but also to use it as a diagnostic tool by boosting the capabilities of existing techniques such as dynamic MRI, functional MRI, or urodynamics. By strengthening prediction models, AI may shortly have significant therapeutic benefits in the field of urogynecology. Clinical practice, on the other hand, has always required physicians to handle huge amounts of data, ranging from histories and physical examinations to laboratory and imaging investigations, and, most recently, genetic data. The capacity to handle this complexity will always distinguish good physicians.

## Author contributions

All authors listed have made a substantial, direct, and intellectual contribution to the work, and approved it for publication.

## Conflict of interest

The authors declare that the research was conducted in the absence of any commercial or financial relationships that could be construed as a potential conflict of interest.

## Publisher’s note

All claims expressed in this article are solely those of the authors and do not necessarily represent those of their affiliated organizations, or those of the publisher, the editors and the reviewers. Any product that may be evaluated in this article, or claim that may be made by its manufacturer, is not guaranteed or endorsed by the publisher.
